# How to Study Materia Medica

**Published:** 1887-12

**Authors:** 


### How to Study Materia Medica. Three Lectures. By Conrad Wesselhœft, M. D. Boston: Otis Clapp & Son, 1887.

The study of the materia medica is a *very* difficult one, and any assistance in it will always be welcome. The late Dr. Carroll Dunham left us a useful essay upon this subject. Dr. Wesselhœft's lectures give some useful hints to help students. But his fault is that he considers pathology the basis of the materia medica; symptoms which have no pathological basis he would ignore; yet many, perhaps most, of our most wonderful cures have been made by the use of these "visionary" symptoms.

